# Epidemiology and prevention of influenza in children in Argentina and Brazil

**DOI:** 10.26633/RPSP.2017.76

**Published:** 2017-04-28

**Authors:** Argentinean Influenza Brazilian Working Group Vaccine

**Affiliations:** 1 Hector Abate, Pediatric Infectious Diseases Division, Hospital Humberto Notti, Mendoza, Argentina; Pablo Bonvehi, Infectious Diseases Division, Centro de Educación Médica e Investigaciones clínicas “Norberto Quirno” (CEMIC), Buenos Aires, Argentina; Ana Ceballos, Pediatric Infectious Diseases, Sociedad Argentina de Pediatría, Córdoba, Argentina; Ralf Clemens, Global Research in Infectious Diseases (GRID), Rio de Janeiro, Brazil; Alejandro Ellis, Pediatric Infectious Diseases, Sociedad Argentina de Pediatría, Buenos Aires, Argentina; Gabriela Ensinck, Pediatric Infectious Diseases Division, Hospital de Niños “Víctor J. Vilela”, Rosario, Argentina; Angela Gentile and Norberto Giglio, Epidemiology and Primary Care Division, Hospital de Niños Ricardo Gutierrez, Buenos Aires, Argentina; Silvia González Ayala, Universidad Nacional de La Plata, La Plata, Argentina; Renato Kfouri, Pediatric Infectious Diseases, Sociedade Brasileira de Imunizações, São Paulo, Brazil; Gabriel Oselka, Universidade de São Paulo, São Paulo, Brazil; Marco Aurélio Palazzi Sáfadi, School of Medicine, Santa Casa de São Paulo, São Paulo, Brazil; Charlotte Russ, Pediatric Infectious Diseases, Sociedad Argentina de Pediatría, Buenos Aires, Argentina; Ricardo W. Rüttimann and Daniel Stamboulian, Fighting Infectious Diseases in Emerging Countries (FIDEC), Miami, Florida, United States of America; and Carla Vizzotti, Sociedad Argentina de Infectología, Buenos Aires, Argentina.

**Keywords:** Orthomyxoviridae, pediatrics, influenza, vaccines, Argentina, Brazil, South America, Orthomyxoviridae, pediatría, vacunas contra la influenza, Argentina;, Brasil, América del Sur, Orthomyxoviridae, pediatria, vacinas contra influenza, Argentina, Brasil, América do Sul

## Abstract

*A group of influenza experts from Argentina and Brazil got together to discuss the burden of influenza in children, review current vaccine coverage rates in both countries, analyze vaccine effectiveness, and discuss strategies to improve prevention. Active surveillance of respiratory viruses is carried out nationwide in both countries. Years 2014 and 2015 were mild influenza seasons; influenza virus type A/H3N2 prevailed, whereas type B represented less than 30% of isolates. Trivalent inactivated influenza vaccine is included in National Immunization Programs for 1) children 6 months to 2 years old in Argentina; 2) children 6 months to 5 years old in Brazil; and 3) all high-risk individuals. Coverage rates in both countries were about 80% (albeit lower for the second dose). Experts from both countries proposed the following strategies to improve prevention: 1) increase surveillance; 2) assess effectiveness and long-term safety of influenza vaccines; 3) reinforce vaccination programs in order to increase coverage rates; and 4)consider introducing more effective vaccines, such as adjuvanted trivalent vaccines. In Argentina, estimating casefatality rates was also recommended. Other proposed actions included enhancing education of health professionals and the lay community, and better use of communication resources to raise awareness of the burden of influenza and promote vaccine uptake*.

## RESUMEN

Expertos en influenza de Argentina y Brasil reunidos en un grupo de trabajo evaluaron la carga de enfermedad de influenza en niños, analizaron las coberturas vacunales, la efectividad de las vacunas y discutieron estrategias para mejorar la prevención. En ambos países se realiza vigilancia de virus respiratorios en todo el territorio. Las últimas temporadas de gripe fueron leves, con predominio de influenza A H3N2, influenza B representó menos del 30% de los aislamientos (con co-circulación de ambos linajes). La vacuna de influenza inactivada trivalente está incluida en el Programa Nacional de Inmunizaciones en niños entre 6 meses-2 años en Argentina y hasta los 5 años en Brasil, y en niños mayores con factores de riesgo. Las coberturas alcanzan 80% (menor para la segunda dosis).

Las estrategias propuestas incluyen incrementar la vigilancia de la enfermedad y estimar la tasa de letalidad (Argentina), realizar vigilancia de efectividad y seguridad de las vacunas, fortalecer los programas para aumentar las coberturas y considerar incorporar nuevas vacunas más eficaces. También se discutieron acciones de educación, tanto en la comunidad como en los trabajadores de la salud, y de comunicación para concientizar sobre el impacto de influenza en la población y la importancia de su prevención.

## RESUMO

Um grupo de especialistas em influenza da Argentina e do Brasil reuniu-se para discutir o ônus da influenza em crianças, analisar os índices de cobertura vacinal nos dois países, analisar a efetividade das vacinas e discutir estratégias para melhorar a prevenção. A vigilância ativa de vírus respiratórios é realizada em âmbito nacional em ambos os países. Nos anos 2014 e 2015, as temporadas de gripe foram leves; o tipo A/H3N2 do vírus da influenza prevaleceu, enquanto que o tipo B representou menos de 30% dos isolados. A vacina trivalente inativada contra a influenza está incluída nos programas nacionais de vacinação para 1) crianças de 6 meses a 2 anos de idade na Argentina; 2) crianças de 6 meses a 5 anos de idade no Brasil; e 3) todos os indivíduos de alto risco. As taxas de cobertura em ambos os países foram de aproximadamente 80% (porém menores para a segunda dose).

Os especialistas de ambos os países propuseram as seguintes estratégias para melhorar a prevenção: 1) aumentar a vigilância; 2) avaliar a efetividade e segurança a longo prazo das vacinas contra a influenza; 3) reforçar os programas de vacinação para aumentar as taxas de cobertura; e 4) considerar a possibilidade de introduzir vacinas mais eficazes, como as vacinas tríplices com adjuvante. Recomendou-se também calcular as taxas de letalidade na Argentina. Outras ações propostas incluíram melhorar a capacitação dos profissionais da saúde e da comunidade leiga e melhorar o uso de recursos em comunicação para aumentar a conscientização sobre o ônus da influenza e promover a vacinação.

Worldwide, influenza causes a substantial health burden in children, particularly infants and young children. In the United States, despite universal influenza immunization, reported hospitalizations rates for children in 2014–2015 were 50–60 per 100 000, and 100–170 influenza-related pediatric deaths occurred that same season (1). Most hospitalizations (80%) were caused by influenza A subtype H3N2 (A/H3N2), but influenza B accounted for one-third of deaths (2).

On November 18, 2015, influenza experts from Argentina and Brazil participated in a roundtable meeting in Rio de Janeiro to 1) assess the burden of influenza illness in children; 2) analyze vaccine coverage and vaccine effectiveness; and 3) discuss strategies to improve prevention. This article summarizes the results of the meeting (“Looking into the Future in the Prevention of Influenza in Children: How Will New Vaccines Provide Better Protection”), which was organized by a nonprofit organization known as FIDEC (Fighting Infectious Diseases in Emerging Countries) (Miami, Florida, United States). The working group of influenza experts discussed influenza epidemiology in Argentina and Brazil, influenza immunization programs, coverage rates, and evidence for vaccine effectiveness. The experts reviewed the rationale for routine immunization in children, discussed challenges in achieving effective control programs, and proposed strategies and actions to improve protection.

## EPIDEMIOLOGY AND BURDEN OF INFLUENZA

### Argentina

In Argentina, influenza surveillance data are provided by the National Laboratory Surveillance System (*Sistema Nacional de Vigilancia de Salud*, SNVS). In 2014, the system reported 2 308 influenza virus isolates (77% type A and 23% type B) (3). Up until epidemiological week 43 of 2015, the system reported 2 049 isolates: 91% type A (52% H3, 10% H1N1pdm09, and 38% not subtyped) and 9% type B (mainly Yamagata) (3).

The burden of influenza can be assessed through acute respiratory infection hospitalizations. At a children’s hospital in Buenos Aires (Hospital de Niños Ricardo Gutierrez), during 2000–2013, influenza accounted for 7% of all respiratory virus isolations (4). In a pharmaco-economic study at the same institution, estimated annual costs attributable to influenza in children under 5 years old ranged from US$ 250 000 to US$ 350 000 (5). Influenza mortality data are not available for the same period for Argentina.

### Brazil

In Brazil, disease surveillance covers influenza-like illness (ILI) (at sentinel sites) and severe acute respiratory syndrome (SARS) (in patients admitted to intensive care units, and in universal SARS surveillance). In 2014–2015, the influenza seasons were mild (6, 7), with type A/H3N2 predominant and late circulation of type B. In 2015, approximately 12 000 ILI samples were processed; 25% were positive for a respiratory virus, and 50% of those were influenza (55% A/H3N2, 30% B, 8% non-subtyped A, and 7% A/H1N1) (7). Influenza was more prevalent in children more than 4 years old, and type B predominated in adolescents. The South and Southeast regions accounted for most of the positive samples, with A/H3N2 prevailing, and co-circulation of A/H3N2, A/H1N1, and B occurring in the South region. Based on the results of the universal SARS surveillance, 8% of 12 300 samples were influenza viruses. In São Paulo, 20% of influenza-related hospitalizations were due to type B (7).

A total of 1 420 SARS-related deaths were reported up to October 2015, with 151 (11%) caused by influenza (45% A/H3N2, 23% B, 17% A/H1N1, and 15% non-subtyped A) (7). The influenza mortality rate was 0.08 per 100 000 population, and 70% had at least one risk factor (age over 60 years, chronic cardiovascular or pulmonary condition, diabetes, or obesity). Only 11% had received a flu vaccine. A/H1N1 had a higher case-fatality rate than other influenza viruses.

In a study of respiratory viral infections in children under 2 years old hospitalized at the Santa Casa Hospital in São Paulo, from 2008 to 2010 (8), 10% of all respiratory isolates were influenza viruses and 50% of children had underlying health conditions.

## ROUTINE IMMUNIZATION, COVERAGE RATES, AND VACCINE EFFECTIVENESS

### Argentina

In Argentina, trivalent inactivated influenza vaccine (IIV3) was introduced into the National Immunization Program (NIP) in 2011 for children 6 months to 2 years old and other target groups including 1) high-risk individuals (e.g., those with chronic respiratory and cardiovascular conditions, diabetes, immunocompromising conditions, etc.); 2) pregnant or postpartum women; 3) health care workers; and 4) adults 65 years old and older (9). From 2011 to 2015, vaccine coverage in young children (6 months to 2 years old) ranged between 72% (for the first dose) and 50% (for the second) (9).

IIV3 effectiveness was assessed through a case-control study carried out at three pediatric hospitals (10). Although the total number of cases was low (38 cases and 92 controls), preliminary effectiveness was 73% in children 6 months to 2 years old. Results from the Pan American Health Organization (PAHO) Network for Evaluation of Influenza Vaccine Effectiveness in the Latin America and Caribbean Region (*Red para la Evaluación de Vacunas En Latino América y el Caribe–influenza*, REVELAC-i) assessing influenza vaccine effectiveness showed a lower protection rate (48%) for the prevention of severe infections in children under 5 years old (11).

Vaccination of pregnant women can help prevent influenza in newborns and young infants (12). In 2015, IIV3 coverage in this population exceeded 90% (3). A study carried out in Argentina using the influenza A/H1N1 MF59-adjuvanted vaccine (13) demonstrated that vaccinated pregnant women had a lower risk of 1) giving birth to low-weight babies (odds ratio (OR): 0.74) and 2) premature deliveries (OR: 0.79). Furthermore, vaccination was not associated with adverse perinatal or maternal events.

### Brazil

In Brazil, influenza immunization is routinely administered to children 6 months to 5 years old, adults 60 years old and older, pregnant or postpartum women, health care workers, the indigenous population, individuals in prison, and high-risk groups (e.g., those with chronic respiratory or cardiovascular diseases, diabetes, immunocompromising conditions, etc.) (14). Overall, in 2011–2014, vaccination coverage exceeded 80% in all groups (15), and in 2015 approximately 50 million people (25% of the population) were immunized (14). Most vaccines used during the 2016 influenza season were manufactured locally by the Instituto Butantan (São Paulo).

## RATIONALE AND HURDLES OF ROUTINE INFLUENZA IMMUNIZATION IN CHILDREN

The burden of influenza in children is substantial and hospitalization rates are highest among the youngest children (16, 17). Notably, half of flu-related hospitalizations and deaths occur in previously healthy children, so a vaccination strategy that only includes children with comorbidities does not seem to be effective (18).

Influenza immunization in children is the most effective way to prevent disease, affording both direct and indirect protection. The protective effect of the vaccine depends largely on the match between vaccine strains and circulating viruses. As children are usually the family members who bring influenza into their household, vaccinating children could help mitigate outbreaks (19). One health care model showed that vaccinating 20% of schoolchildren had a greater impact on flu-related mortality than vaccinating 90% of the elderly (20). When influenza immunization of schoolchildren was introduced in Japan, all-cause deaths, as well as influenza and pneumonia-related deaths, were significantly reduced in all age groups (21), and when vaccination was stopped, mortality rates increased, especially in the elderly.

There are, however, several hurdles to introducing an early childhood influenza control program. First, there are limited vaccine options. IIV3s have moderate efficacy in young children (22), and do not induce persistent immune response. Inactivated influenza vaccines, quadrivalent (IIV4s) offer broader protection against the two B lineages, which usually co-circulate yet alternate in dominance (2). Although B viruses predominate in children, they are a significant cause of hospitalizations and deaths in all age groups. Use of IIV4 could reduce a mismatch between a vaccine and circulating B lineages, but the benefits would be modest (23). Adjuvanted IIV3s (aIIV3s) are more effective than non-adjuvanted vaccines. In a trial of MF59-adjuvanted IIV3 in 4 700 children 6–72 months old (24), efficacy was 85% (the highest ever reported in children 6–24 months old) and persisted after the second dose, and there were no safety issues. According to one study, the correlate of protection threshold is higher in children than in adults (25); for example, titers of 1:110 in children and 1:40 in adults were both associated with 50% clinical protection. In Canada, aIIV3 is licensed for use in infants and young children 6–24 months old due to its superior immunogenicity and acceptable safety profile (26). At the roundtable meeting, use of the live attenuated vaccine was also discussed, but as this vaccine is not available in Argentina or Brazil, the information was not included in this report.

A second hurdle to introducing a childhood influenza control program is related to safety and reduced confidence due to adverse events following immunization (AEFIs), a problem mostly seen in Europe and North America. Unexpected AEFIs with two flu vaccines have had a negative impact (27, 28). In addition, poor vaccine performance could discredit the integrity of the NIP and dampen the success of other routine vaccines.

A third hurdle concerns funding requirements. Introducing flu vaccine into an NIP requires evidence of cost-effectiveness involving high vaccine efficacy; reasonable cost (with drawbacks including the need for two-dose priming and/or annual revaccination); and negligible AEFI costs. Currently, 29 countries in Latin American but only seven in Europe have routine influenza childhood immunization programs, and vaccine uptake in developed countries does not surpass 30%.

At the meeting, various solutions were suggested to overcome these hurdles, including 1) using more efficacious vaccines; 2) building greater confidence in safety by extending post-licensure surveillance; and 3) making public programs more flexible by broadening the interval of the two-dose schedule, irrespective of the season, and expanding school delivery programs to include children 1–5 years old.

## CHALLENGES IN INFLUENZA PREVENTION IN ARGENTINA AND BRAZIL

The meeting participants agreed that assessing the burden of disease and the impact of vaccination is more difficult for influenza than for other vaccinepreventable diseases because influenza cannot be eradicated, symptoms are nonspecific, cases are not usually virologically tested, and herd protection is difficult to measure.

Immunization rates are decreasing in Argentina and Brazil (9, 15) for flu as well as other vaccines, although both countries still retain the highest influenza vaccination rates worldwide. One reason for this downward trend is parent misinformation. Parents often see influenza as a mild disease and thus view the influenza-like symptoms from the vaccines as outweighing any benefits (29). In addition, people get tired of having to get shots every year. The growing influence of local anti-vaccination advocacy groups is also a cause for concern.

Another factor in the lower coverage is the logistics related to limited staffing for administering routine vaccines in a crowded childhood immunization calendar.

Education of health care workers is another problem that needs to be tackled. As shown in a study evaluating missed opportunities for flu vaccination, from the parents’ perspective (30), the main reason for the lower coverage was a lack of information from health care workers, who were not recommending the vaccine. In Argentina, few specialists recommend influenza vaccination for children with chronic comorbidities at highrisk for flu-related complications.

In discussing the best vaccine options, the meeting participants emphasized the superiority of the aIIV3 compared to the IIV4. While the second B strain may add 15% efficacy, leading to an overall IIV4 efficacy of 60%–65%, the efficacy of adjuvanted vaccines exceeds 80%, while also providing protection for mismatched B lineage. In Brazil, although the IIV4 became available in the private market in 2015, the Ministry of Health is not considering incorporating it into the NIP in the near future.

Safety concerns for use of repetitive doses of adjuvanted vaccines in young children were also addressed. Canada has licensed the aIIV3 with limited indication in the youngest age group (6–24 months) (26). In Argentina, the national immunization committee has resolved to continue using IIV3 and will consider incorporating adjuvanted vaccines in the future (a technology transfer agreement will enable local vaccine production).

## STRATEGIES AND ACTIONS TO IMPROVE PROTECTION

Following the roundtable meeting discussions the participants proposed strategies to improve disease surveillance and actions to increase protection in Argentina and Brazil.

### Surveillance strategies

Recommended surveillance strategies included 1) developing an influenza monitoring system and unified national database (both countries) and 2) estimating case-fatality rates (in Argentina only).

### Protection actions

Recommended protective actions were grouped into three categories: 1) vaccination, 2) education, and 3) communication. Proposed vaccination actions included 1) conducting continued surveillance of effectiveness of inactivated vaccines; 2) estimating coverage in different populations; 3) increasing vaccination in pregnant women for protection of young infants; 4) promoting vaccination at childcare centers; 5) carrying out longterm vaccine safety surveillance to avoid AEFIs; 6) considering the introduction of aIIV3s for infants and children; and 7) considering universal vaccination of schoolchildren to gain herd protection (in Brazil; in Argentina, this strategy was not deemed feasible in the short-term because the main objective there is to reduce morbidity and mortality in high-risk groups rather than reducing viral circulation). Proposed education actions included: 1) enhancing education of health professionals; 2) reinforcing the nurse’s role in promoting immunization and delivering vaccines; 3) raising awareness of the risk of influenza in the population; 4) targeting high-risk groups, working with scientific societies and specialists; and 5) strengthening the physician–parent relationship and helping parents understand the risk/benefits of vaccines. In the same vein, the following communication activities were suggested to raise awareness of the value of vaccines: 1) using media to communicate the risks of vaccine-preventable diseases; 2) using the Internet and social media to promote vaccine uptake; and 3) using reminder cards for vaccination schedules.

## CONCLUSIONS

The high burden of influenza in children in Argentina and Brazil calls for sustained efforts to improve protective measures. There is a need for more effective flu vaccines for infants and young children. Surveillance programs should continue to 1) monitor for changes in circulating viruses and 2) assess vaccine effectiveness and safety. Increasing vaccine coverage levels, introducing adjuvanted vaccines, and continuing the development of more effective vaccines are all goals that should be pursued.

### Funding.

Novartis Vaccines (Cambridge, Massachusetts) provided financial support for the meeting.

### Disclaimer.

Authors hold sole responsibility for the views expressed in the manuscript, which may not necessarily reflect the opinion or policy of the *RPSP/PAJPH* or the Pan American Health Organization (PAHO).
